# Monkeypox Proctitis: A Case of Targeted Treatment With Tecovirimat

**DOI:** 10.7759/cureus.36238

**Published:** 2023-03-16

**Authors:** Premalkumar Patel, Cynthia Espinosa, Varsha Konyala, Nicholas S Camps, Estafenia Cecilio, Aakangsha Jain, Claudio Tuda

**Affiliations:** 1 Infectious Disease, Mount Sinai Medical Center, Miami Beach, USA; 2 Internal Medicine, Mount Sinai Medical Center, Miami Beach, USA; 3 Infectious Diseases, Mount Sinai Medical Center, Miami Beach, USA; 4 Osteopathic Medicine, Nova Southeastern University Dr. Kiran C. Patel College of Osteopathic Medicine, Fort Lauderdale, USA

**Keywords:** proctitis, muscosal lesion, anogenital lesion, hiv, tecovirimat, monkeypox virus

## Abstract

Monkeypox (MPX) is an exanthematous disease first identified in the 1950s, associated with animals in Central and Western Africa, and has since been found sporadically worldwide. In May 2022, a returning family from Nigeria tested positive for MPX, which marked the onset of the current outbreak.^ ^It has now become a disease of concern in most parts of the world. The current standings are nearing 90,000 cases, with numbers increasing daily. The United States reported 29,711 cases so far.^ ^The characteristic exanthem of MPX is known to be present ubiquitously on the human habitus, with recent reports describing anogenital and mucosal lesions. Here, we present a rare case of a 43-year-old male presenting with excruciating perianal pain and purulent discharge, found to have proctitis secondary to MPX, and subsequently treated with Tecovirimat, a targeted antiviral therapy.

## Introduction

Monkeypox (MPX), an orthopoxvirus, is genetically similar to the smallpox virus [1,2,3]. It has come to the forefront in recent times due to pockets of infections in countries that have never historically reported them. In the current outbreak, MPX has frequently been identified in men who have sex with men (MSM). A patient may present with skin lesions located principally on the face, trunk, arms, and legs. This can also be associated with flu-like symptoms such as fever, chills, headache, sore throat, myalgia, pruritus, fatigue, and lymphadenopathy. Compared to MPX cases in the past, current cases report that these patients have also been presenting with genital ulcers with involvement of the anogenital mucosa [2,3,4]. Naturally, this form of presentation can resemble sexually transmitted infections (STIs), which could lead to clinician bias and misdiagnosis. In the United States, as per the Centers for Disease Control and Prevention (CDC) recommendation, polymerase chain reaction (PCR) testing for orthopoxvirus DNA from the lesion samples is widely used for diagnosing MPX. The management of MPX virus infection ranges from supportive care to antiviral therapy based on the severity of the disease process. Here, we present a unique case of MPX with painful perianal and rectal lesions in an immunocompetent patient, successfully treated with the investigational therapy antiviral, Tecovirimat.

## Case presentation

A 43-year-old African American male patient with a history of the human immunodeficiency virus (HIV), well-controlled on antiretroviral treatment for the past 15 years (CD4 percentage = 25.05%), was admitted to the hospital for perianal lesions, draining purulent discharge, rectal pain, left groin pain, and maculopapular-pustular lesions on the body associated with fever, chills, headache, and fatigue.

The patient was in a good state of health until five days before the admission when he began to develop three perianal lesions accompanied by draining mucopurulent discharge and progressive 9/10 rectal pain. His associated symptoms included fever, chills, generalized malaise, fatigue, headache, and a macular rash on the palms followed by papular and pustular lesions on his face, chest, back, and upper and lower limbs. There were no subjective reports of pruritus or bowel habit changes. The patient reported being a male who engaged in sexual activities with men for over a year and confirmed unprotected sexual intercourse with two known partners in the past six months. He had a notable medical history of primary syphilis and genital herpes simplex virus (HSV). He was a current cigarette smoker, with 0.75 pack years, a reported *social drinker*, and smoked marijuana occasionally. He was a chef’s assistant by occupation and had had no recent travel history, hospitalizations, or exposure to animals or sick contacts.

**Figure 1:**
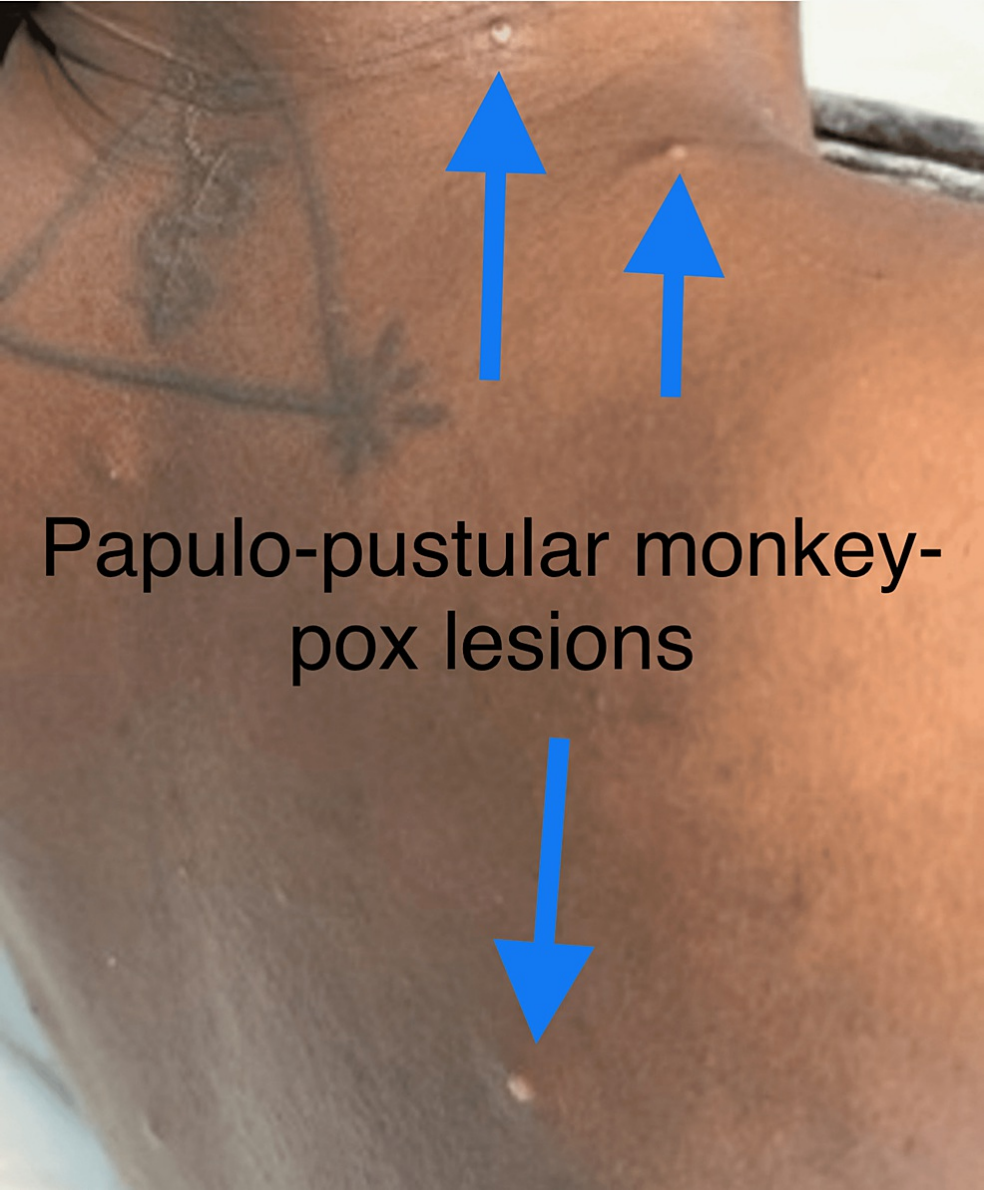
Papulopustular lesions on the back.

His physical examination revealed an ill-appearing man who was diaphoretic with a temperature of 103 °F, normotensive, tachycardia at 109 beats per minute, and a respiratory rate of 18 cycles per minute with saturation at 94% on room air. There was significant anterior cervical lymphadenopathy and tenderness, three oral shallow ulcers on the soft and hard palate, and a diffuse and scattered maculopapular rash on the palmar aspect of both hands. There were approximately 15 papulopustular lesions with surrounding erythema (2-4 mm in size) scattered on the face, back, and chest (Figure 1).

**Figure 2:**
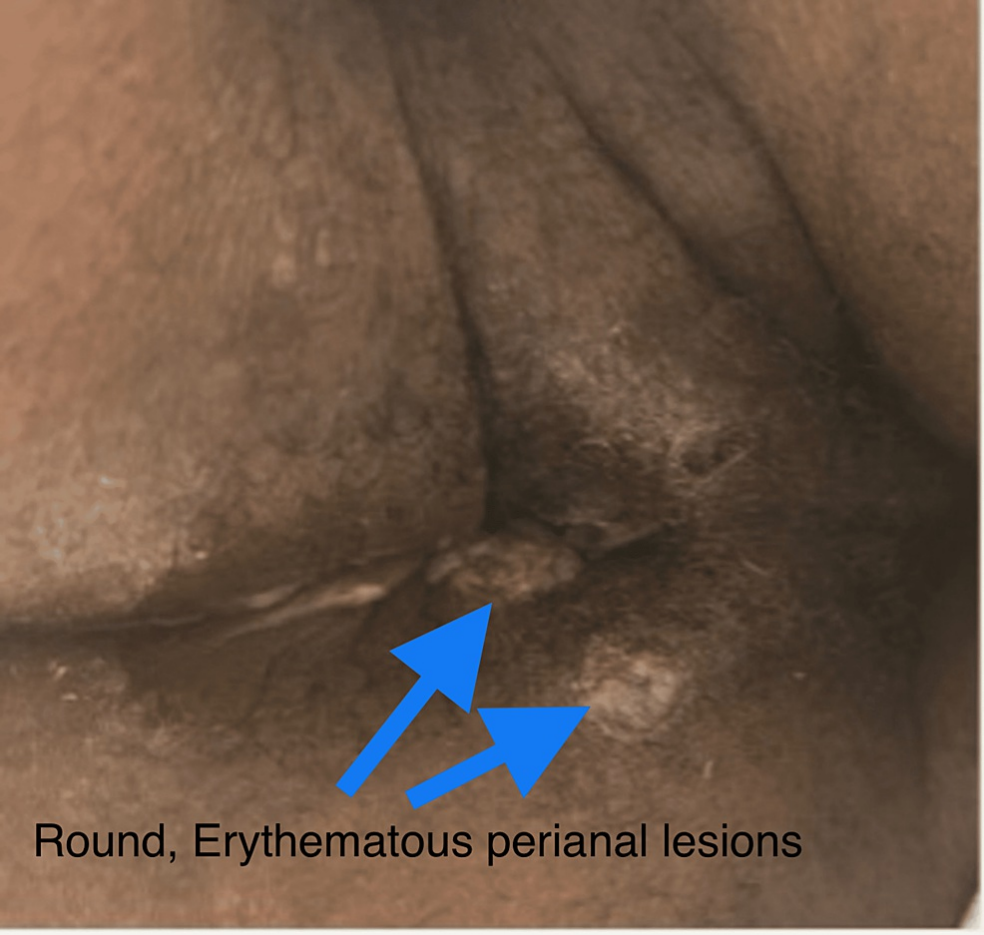
Perianal lesions.

**Figure 3:**
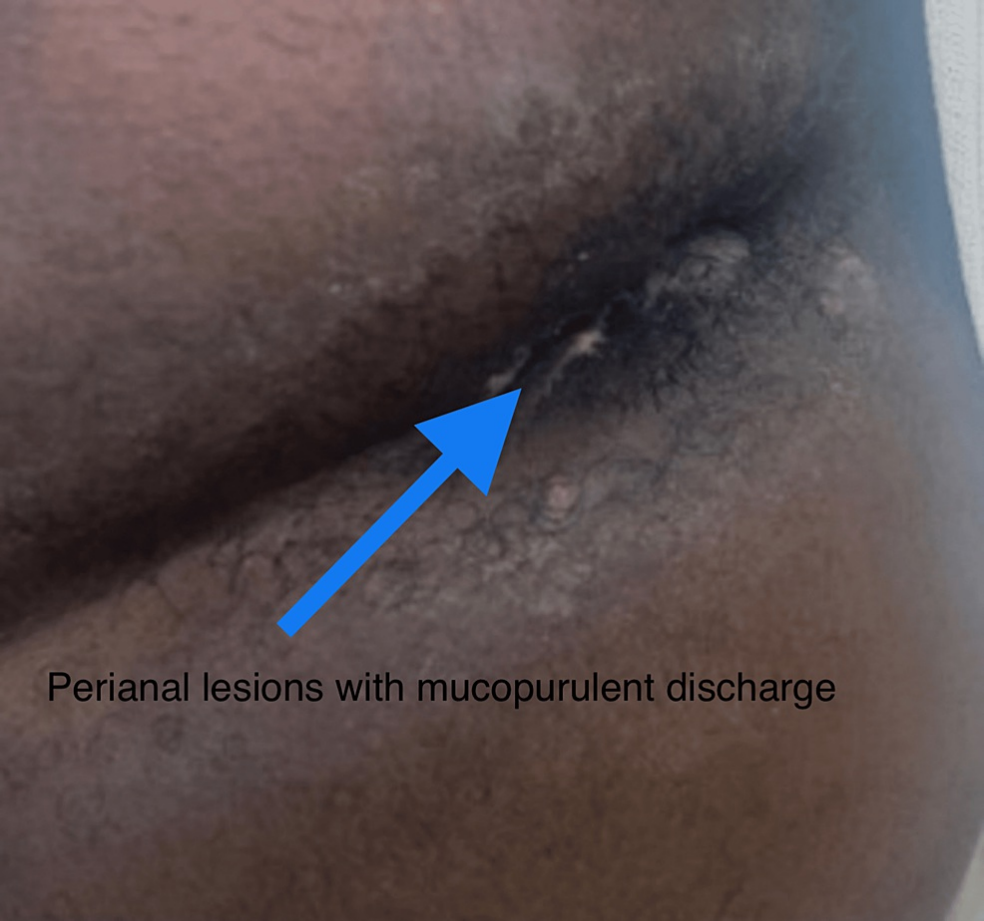
Perianal lesions with mucopurulent discharge.

Three 1 cm perianal lesions were identified, one of which appeared round, erythematous, and draining mucopurulent discharge (Figures 2-3).

 

**Figure 4:**
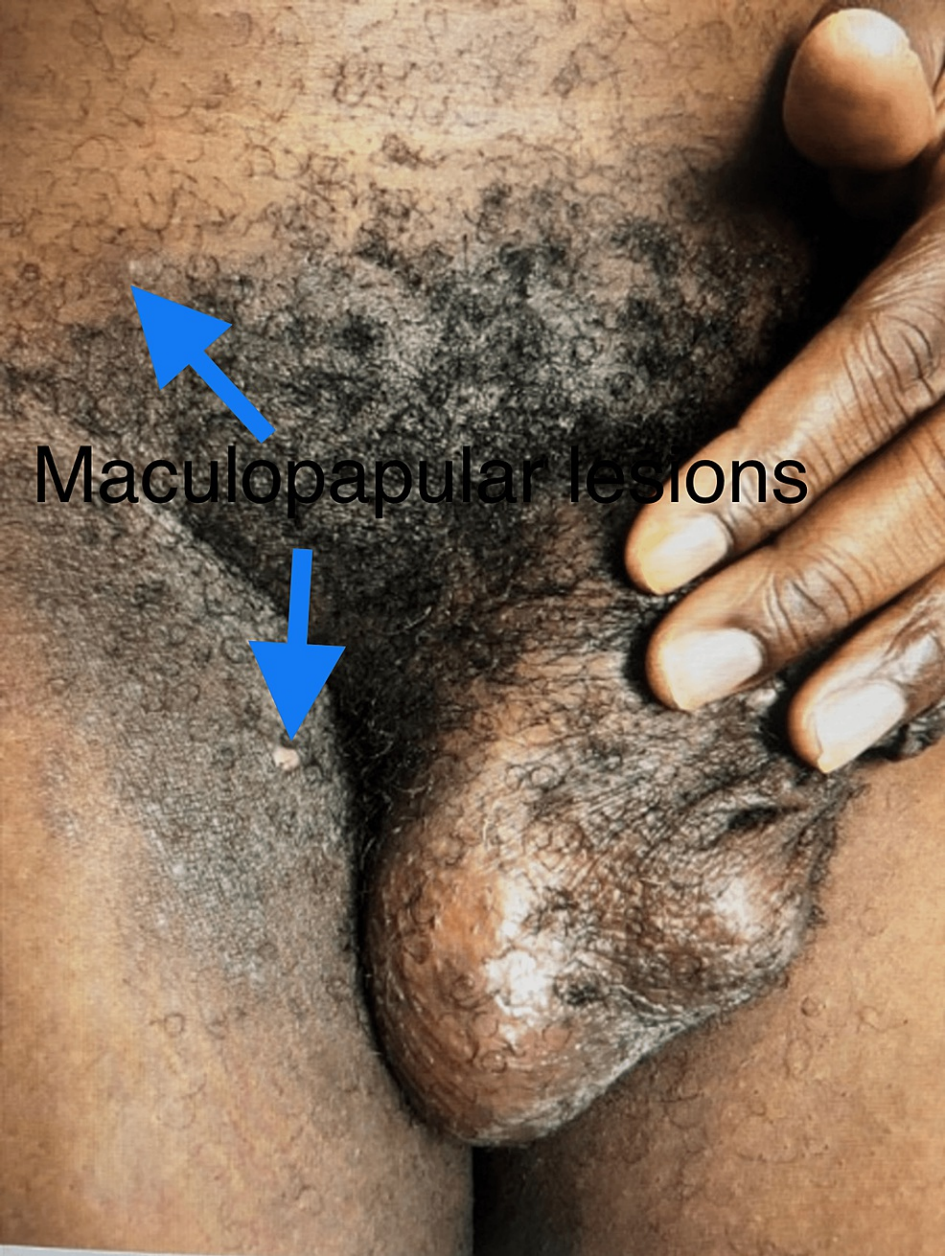
Maculopapular lesions surrounding the genital area.

The remainder of the lesions were round, firm, shallow, intact, and tender vesicles without discharge. Prominent painful left inguinal lymphadenopathy was also noted (Figure 4).

Preliminary blood tests showed atypical lymphocytosis, suggesting an ongoing viral infective process. Normal CD4 counts at 867 cells/mm^3^ (CD4/CD8 0.66 cells/mm^3^) and CD4 percentage at 25% suggested a stable immunologic status. The metabolic panel was significant for an increase in creatinine at 1.54 mg/dL. Rapid plasma reagin was reactive at 1:128 dilution.

**Figure 5:**
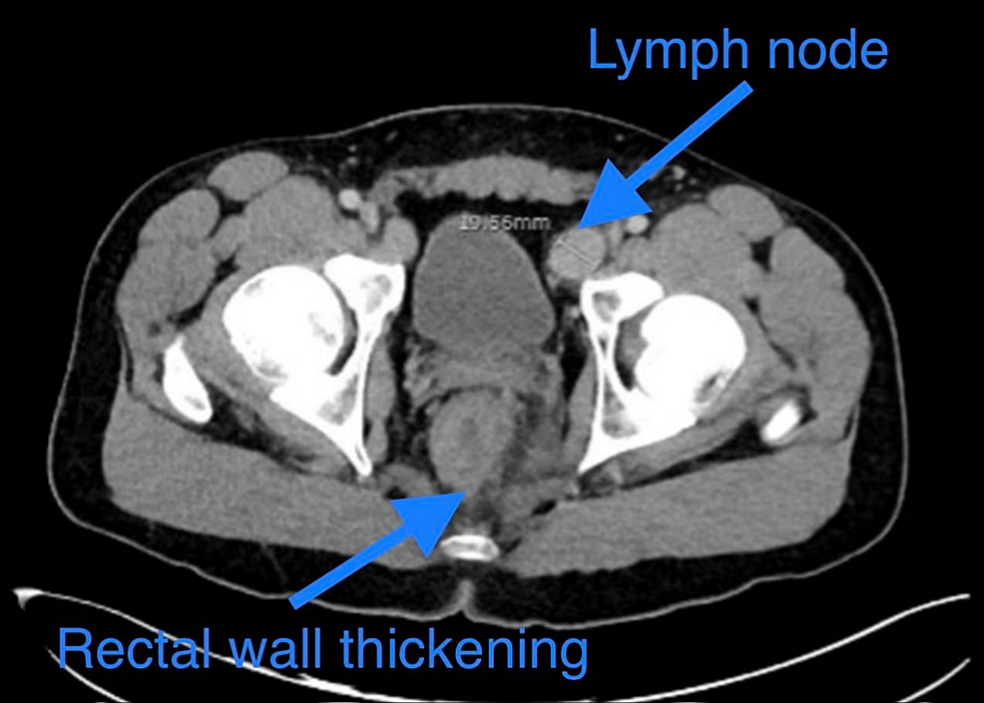
CT abdomen and pelvis depicting rectal thickening and an enlarged lymph node. CT, computed tomography

The patient was empirically started on doxycycline 100 mg two times a day and valacyclovir 1 g three times a day and simultaneously treated with intramuscular benzathine penicillin G 1.2 million IU once for secondary syphilis. Rectal swabs obtained for chlamydia, gonorrhea, and HSV were negative. Blood culture samples collected were unremarkable. Computed tomography (CT) abdomen and pelvis with contrast showed bilateral inguinal and iliac chain lymphadenopathy measuring up to 23 mm in the left inguinal region and 20 mm in the left external iliac chain. Additionally, diffuse circumferential rectal wall thickening, edema with significant mesorectal fat stranding, and lymph nodes measuring about 10 mm were present, signaling an infectious proctitis (Figure 5).

Over the next two to three days, the patient continued to have episodes of fever, chills, myalgias, and persistent rectal pain with mucopurulent discharge. The patient also complained of fatigue, throat pain, and dysphagia. Additionally, the papulopustular lesions increased in number, with newly appearing lesions in the genital and facial areas. These were accompanied by an increasing white blood cell count from 10,060 to 12,089 cells/mm^3^. A negative monospot test result ruled out infectious mononucleosis. An MPX PCR was ordered, given the clinical picture - rapidly evolving lesions with umbilication in a similar stage of development with diffuse lymphadenopathy. Airborne and contact precautions were instituted. Local care for the rectal pain and surrounding lesions was provided with a sitz bath and lidocaine. The MPX PCR test with swabs taken from two different skin lesions came back positive on day 6 of admission. The clinical presentation appeared to be an aberrant infection with involvement of a protected site, the rectum, in a controlled HIV-positive patient, which prompted us to consider treatment with Tecovirimat 600 mg orally, twice daily, for 14 days based on the CDC guidelines. The State Department of Public Health was notified, and surveillance was conducted. The patient was seen at an outpatient clinic on day 10 of antiviral use with almost complete resolution of symptoms.

## Discussion

MPX is a large DNA virus and is grouped with other viruses with similar antigenicity and structural functional traits under the umbrella of *poxviruses*. Due to these overlapping traits, a similar clinical course in the disease activity of all these viruses is expected. This cross-reactivity also justifies the waning vaccinia-conferred immunity against smallpox and a significant rise in MPX cases.

MPX is largely transmitted via respiratory secretions as well as close contact with infectious skin lesions and fomites. MPX infection has an incubation period of five to 21 days followed by symptoms of fever, a characteristic rash, and significant lymphadenopathy. The rash has been described as multiple papules, pustules, and even ulcerative lesions, more so on the face and extremities. Mucosal lesions involving the rectum and pharynx are seldom observed [1].

A study by Thornhill et al. [2] reported that 99% of the infections were seen in males with a median age of 38 years, with 98% being either homosexual or bisexual men. In these patients, there was a notable prevalence of HIV. While skin lesions were noted in nearly all the patients, the most common site involved was the anogenital area followed by the face and extremities. There were a sizeable number of patients who presented with only a single ulcerative lesion in the genital area, which may masquerade as an STI. Mucosal lesions as well have been commonly observed; the rectum, buccal cavity, and conjunctiva presented with proctitis or change in bowel habits, inflamed pharynx or tonsils, or conjunctivitis, respectively. Flu-like symptoms such as fever, lethargy, myalgias, and headache have been the other symptoms reported. Given the disproportionate cases involving the anogenital area and rectum, it is important to elicit sexual history. A large number of patients with infections have been reported to have had intimate, sexual contact in the recent past [2,3]. While there may be no definite need for hospital admission, the most common causes for inpatient care involved severe anorectal pain, inability to consume food orally, eye lesions, or acute kidney injury.

The diagnosis of MPX can be a challenge as most patients present with lesions that mimic other well-known diseases such as smallpox and varicella. The presence of anogenital-only lesions or throat involvement with lymphadenopathy can raise suspicion for STIs and infectious mononucleosis. Testing for STIs, as we did particularly for HIV, chlamydia, gonorrhea, HSV, and Epstein-Barr viruses, may also assist in clinching the diagnosis.

While ruling out these diseases is important, it is also paramount to diagnose MPX swiftly. A quick diagnosis can facilitate the implementation of infection control and strict precautionary measures. Currently, PCR is the diagnostic test of choice to identify MPX viral DNA in humans. Skin lesional swabs and anogenital swabs are the most common specimen collection sites, with urine and seminal secretions being less commonly used.

MPX is expected to follow a self-limiting, mild disease course. Management, therefore, involves symptom alleviation and maintenance of adequate nutrition, hydration, and pain control. Treating secondary bacterial infections and preventing complications are additional measures. However, comorbidities and immunological health are a few of the factors that influence the prognosis and necessitate targeted treatment. The CDC recommends consideration of antiviral drugs such as Tecovirimat in individuals with severe disease, immunocompromised state, the pediatric population aged <8 years, and pregnant and breastfeeding women as well as in individuals presenting with aberrant MPX lesions such as the genitals, anus, eye, mouth, etc. [5,6]. Tecovirimat or TPOXX was a drug first identified in 2005 and has demonstrated efficacy against orthopoxviruses. It targets the gene responsible for the extracellular production of viral particles. It is effective against MPX cases with no major side effects [3,5,7,8]. Our patient showed similar evidence of tolerability and effectiveness of Tecovirimat. Cidofovir and Brincidofovir have also been reported to work against orthopoxviruses in vitro with unclear evidence of their effectiveness in MPX cases.

## Conclusions

The MPX illness can be approached in a multipronged way. Health promotion such as general awareness, education on safe sexual practices, hand hygiene, and precautionary measures while traveling are all examples of good health maintenance before contracting the illness. MPX has been known to present in various ways. An atypical rash, genital pain, lesions, and travel to an endemic area are all indicators that prompt a low threshold to suspect MPX. The same holds for high-risk individuals. Once identified, patients must quarantine for 14 to 21 days. Close contacts must also be isolated for a similar duration. MPX in this outbreak has favored MSM individuals. However, a sexual mode of spread is yet to be ascertained. Therefore, it is imperative to dissolve any stigma to improve health outcomes. This case report also serves as evidence of the tolerability and effectiveness of Tecovirimat, which was used by the CDC guidelines for this disease. In other words, a global effort of the utmost vigilance, surveillance, tracking, testing, isolation, education, symptom alleviation, and treatment is the need of the hour to curb the spread of this exanthematous illness.
